# Consumer Preferences for Cured Meat Products from the Autochthonous Black Slavonian Pig

**DOI:** 10.3390/foods12193666

**Published:** 2023-10-05

**Authors:** Sanja Jelić Milković, Ana Crnčan, Jelena Kristić, Igor Kralik, Ivona Djurkin Kušec, Kristina Gvozdanović, Goran Kušec, Zlata Kralik, Ružica Lončarić

**Affiliations:** Faculty of Agrobiotechnical Sciences Osijek, Josip Juraj Strossmayer University of Osijek, Vladimira Preloga 1, 31000 Osijek, Croatia; sanja.jelic@fazos.hr (S.J.M.); jelena.kristic@fazos.hr (J.K.); igor.kralik@fazos.hr (I.K.); ivona.durkin@fazos.hr (I.D.K.); kristina.gvozdanovic@fazos.hr (K.G.); goran.kusec@fazos.hr (G.K.); zlata.kralik@fazos.hr (Z.K.); ruzica.loncaric@fazos.hr (R.L.)

**Keywords:** cured meat products, Black Slavonian Pig, consumer behavior, intrinsic and extrinsic cues

## Abstract

This study investigated the attitudes of Croatian consumers regarding their preferences for cured meat products from the Black Slavonian Pig. The survey was conducted on a sample of 410 consumers who completed an online survey about their consumption habits, knowledge about the pig breed, and socio-demographic characteristics. In this study, the independent samples *t*-test and ANOVA were conducted to determine the significant differences between the most important attributes in the purchase of cured Black Slavonian Pig products and the socio-demographic characteristics, consumption, and purchase habits of the respondents. Statistically significant differences were found between the studied intrinsic (color, odor, fat content, and salt content) and extrinsic variables (production method, brand, quality mark, and origin) in relation to the socio-demographic characteristics of the respondents (gender, age, employment status, number of household members, and number of children under 15 in the household) and place of purchase, knowledge about the breed, and frequency of consumption of Black Slavonian Pig meat and products. Principal component analysis (PCA) and cluster analysis (CA) were used to determine the consumer segments. Based on the responses received, three homogeneous consumer segments were identified: intrinsically oriented meat consumers, extrinsically oriented meat consumers, and uninterested meat consumers. The information obtained in the study is important for producers and retailers, as they can use our findings to develop successful marketing tools and different marketing strategies to promote cured Black Slavonian Pig products.

## 1. Introduction

Pork is one of the most commonly consumed meats in the world. The consumption of pork has played a crucial role in human evolution and is now an important part of a balanced diet [1,2]. Mankind began producing meat products with the aim of extending shelf life and improving taste. Meat was transformed into different products through salting, curing, and smoking, and today there is a wide range of products that differ from each other in terms of meat type, salt and fat content, processing method, and consumption occasion [1,3,4]. Traditional food products represent regional identity, traditional production methods, and gastronomic heritage [1,4,5,6]. Traditional food products can be a way to preserve autochthonous pig breeds and contribute to the development of sustainability, rural area preservation, and product differentiation to provide consumers with a wide range of food choices [1]. Black Slavonian Pigs have been reared for centuries in the Republic of Croatia. The Black Slavonian Pig, also known as Fajferica, was developed by the earl Karl Pfeiffer in the second half of the 19th century in Slavonia [7]. Pfeiffer created the breed with more desirable economic characteristics (feed conversion ratio, daily gain, carcass traits) compared to breeds in use at the time. The breed was economically successful, known for its fat and meat production, and was one of the most common in the 1950s. Since then, it has been slowly replaced by modern pig breeds, and the population of the Black Slavonian Pig declined drastically in the early 1990s [7]. Nevertheless, the population of the Black Slavonian Pig has increased in the last decade due to conservation measures. According to the data, the number of sows of the breed has increased by 1852 sows in the last ten years (2010–2020), and in 2020 there were 2708 animals. The number of boars increased by 127 animals from 2010 to 2017, but then decreased to 182 animals from 2017 to 2020 [8,9,10,11]. Although the Black Slavonian Pig is under protection measures, wider use of this breed is needed. The Black Slavonian Pig is a fatty-lean type of pig usually used for the production of lard and bacon but also for traditional high-quality meat products [12]. Compared to the meat of modern cosmopolitan breeds, the Black Slavonian Pig has a much higher intramuscular fat content and is also darker and redder than the meat of modern pig breeds [5,13,14,15,16,17]. The production of products of animal origin raises concerns about intensive pig production systems, but the production of traditional food products is usually based on local resources and therefore plays an important role in preserving the natural environment and autochthonous pig breeds [2,4]. The Black Slavonian Pig is traditionally reared outdoors on grassland (including the use of natural resources such as pastures and oaks) or semi-outdoors [12,13]. Rearing pigs in the traditional free-range system is cost- and labor-efficient, as well as environmentally and animal friendly, and results in better meat quality traits and nutritional characteristics [12,13,15,18,19,20]. According to Karolyi et al. [13], Croatian consumers consider Black Slavonian Pig meat an organic product and consider organic products to be very healthy, of high quality, and tasty. Accordingly, meat and products from local breeds, such as the Black Slavonian Pig, have a good image among the public and the media. Nevertheless, consumer attitudes towards fresh meat from the Black Slavonian Pig have not yet been sufficiently studied. Studies show that price, freshness, origin, color, especially for red meat, and fat content are important for consumers when choosing pork, together with quality attributes such as the organoleptic properties of the meat (appearance, tenderness, juiciness, aroma, and taste) and the nutritional quality of the meat [21,22]. Cues can be intrinsic or extrinsic. Intrinsic cues refer to physical aspects of the product (color, shape, appearance), while extrinsic cues refer to non-physical parts of the product (brand, quality, stamp, origin, shop, packaging, production information, etc.) [23,24]. In our study, we investigated the stated importance of nine different product attributes towards cured meat products from Black Slavonian Pig. The aim of this study was to identify consumer segments for cured meat products from Black Slavonian Pigs based on their socio-demographic characteristics, purchasing and consumption habits, and knowledge about the pig breed. The information obtained in the study is important for producers, retailers, and policymakers as they can use our findings to develop successful marketing tools to promote Black Slavonian Pig-cured meat products. 

## 2. Materials and Methods

### Survey Procedure and Statistical Analysis

The survey was conducted among consumers who are older than 18 years, consume pork, and are responsible for purchasing for their household. The survey area was the Republic of Croatia. The survey was conducted using online software because of its advantages over face-to-face, telephone, or postal data collection, such as a larger geographical area, quick response time, lower costs, and fewer errors [25,26,27]. Participants were recruited from the Qualtrics survey panel in June 2021 and distributed by the online survey company Qualtrics Inc. The following criteria were used to select consumers: gender, age, and geographical region of the Croatian population. The number of respondents was previously determined with the Qualtrics Inc. company to represent the Republic of Croatia. Consumers were randomly invited to participate in a closed panel until the quotas for age, gender, and geographical regions representing the general population were reached. A total of 410 Croatian consumers correctly completed the survey, reflecting a representative sample size for the Republic of Croatia with a confidence level of 95.0%.

The collected data were analyzed using the statistical software package IBM SPSS Statistics V26. Data were analyzed using descriptive statistics, non-parametric tests (chi-square χ^2^ test), parametric tests (independent samples *t*–test, one-way analysis ANOVA), and multivariate analysis (factor and cluster analysis, discriminant analysis). Descriptive statistical analysis was used to describe the socio-demographic characteristics of the sample, the consumption and purchasing habits of Croatian consumers, and opinions about the most important attributes when purchasing cured products from the Black Slavonian Pig. The *t*-test for independent samples and ANOVA were conducted to determine the significant differences between the most important attributes in the purchase of cured Black Slavonian Pig products and the socio-demographic characteristics as well as the consumption and purchasing habits of the respondents. Principal component analysis (PCA) and cluster analysis (CA) were used to determine the consumer profiles. Factor analysis made it possible to reduce the number of consumer attitude variables to a smaller set of uncorrelated principal component factors. Cluster analysis was performed on the extracted factor scores from the principal component analysis to group consumers with similar attitudes [28]. To examine the suitability of each intercorrelation matrix for factor analysis, we performed the Kaiser–Meyer–Olkin measure of sampling adequacy (KMO) and Bartlett’s test of sphericity. The extraction method used in the factor analysis was principal component analysis, and we used factors with an eigenvalue greater than 1. Varimax rotation was performed to facilitate the interpretation of each factor. To check the reliability of the factor analysis, Cronbach’s alpha was calculated. Subsequently, the factor scores were used to identify and describe the consumer segments in the cluster analysis. K-means cluster analysis was used to identify different clusters for cured Black Slavonian Pig products based on 410 valid cases. Canonical discriminant analysis was applied to validate the cluster differentiation. The chi-square (χ^2^) test was performed to identify the significant differences between the segments according to consumers’ socio-demographic characteristics, their consumption and purchasing habits, and their knowledge about the origin of the meat. 

## 3. Results and Discussion

### 3.1. Sample Description

A total of 410 respondents completed the online survey; selected socio-demographic variables are listed in Table 1. The sample was composed of 51.0% female and 49.0% male respondents. Overall, 41% of respondents were between 25 and 44 years old, and 26.8% of respondents were 55 or older. Respondents were evenly distributed by geographical regions of the Republic of Croatia. The majority of respondents are employed (in the private, public, or civil sector) (62.0%), and 14.9% of them are retired. Respondents mostly live in families with 2–4 household members (82.7%) and have no children under 15 years of age (68.8%) in the household. In this study, the age limit for children in the household is 15 years, since primary school years are over in the Republic of Croatia and parents have more influence on children’s dietary habits during this period. This is in line with the studies by Smith et al. [29], Pettigrew et al. [30], and Tarabashkina et al. [31], who investigated how the number of children and the age of the children affect parents’ purchasing behavior and whether they have more influence on parents’ purchasing behavior as the age and number of children increase. There is evidence that food marketing influences nutrition knowledge, perceptions, preferences, purchases, and food intake among younger children (under 12 to under 15 years of age), and it is assumed that adults have higher nutrition knowledge. Respondents indicate that their monthly household income group is average (58.8%). The survey also asks consumers about the frequency of their consumption of fresh meat and cured meat products. A total of 25.1% of respondents consume meat four times a week, of which the most common is pork (30.2%) twice a week and cured meat products (19.8%) three times a week. Respondents buy cured pork products mostly in supermarkets (44.6%) or butchers (48.0%). Consumers aged 35–44 buy fresh or cured meat products directly from the producer, while older consumers aged 45–54 and over 55 buy them from butchers or supermarkets (χ^2^ = 17.967, df = 8, *p* < 0.05). Consumers from Eastern Croatia, which is one of the traditional breeding regions of the Black Slavonian Pig, also buy meat directly from the producer, while consumers from north-western and central Croatia buy fresh or cured meat products from butchers or supermarkets (χ^2^ = 17.754, df = 8, *p* < 0.05). The results are consistent with those of Verbeke et al. [32], who concluded that almost half of the Europeans studied in five countries (Belgium, Germany, Poland, Greece, and Denmark) bought fresh or chilled pork and pork products from supermarkets and half from the local butcher, although the distribution varied between countries. The same results were found by Razmaitė et al. [1]. Supermarkets in Lithuania, as in Croatia, are a popular destination for buying pork, as there are not many specialized butchers. A total of 13.2% of the respondents in their study buy pork from butchers, and 17.6% buy it directly from the producer. In the survey, consumers state that they do not know (82.2%) which breed of pig the meat they buy comes from and that they occasionally (55.1%) consume fresh or cured meat products from the Black Slavonian Pig. When asked about the main reason why they do not consume fresh meat or cured meat products from the Black Slavonian Pig or consume it only occasionally, they answered that they have not had the opportunity to taste it (18.5%); it is not easy to find (38.9%); it is expensive (31.3%), and as other reasons, they mentioned that the taste of the meat is different from the meat from hybrid pigs. In addition, the majority of respondents said that there is a lack of information and labels on the product about the breed of pig the meat comes from, so they do not know which breed of pig they are consuming. Younger consumers aged 18–24 and 25–34 say that fresh or cured Black Slavonian Pig meat is not easy to find and they have not had the opportunity to try it, while consumers aged 35–55 believe it is too expensive and contains too much fat (χ^2^ = 38.821, df = 20, *p* < 0.01). Consumers in Spain share the opinion of Croatian consumers about the taste of meat from native breeds of pigs, and they also consider Iberian meat products to be too expensive. According to García-Gudiño et al. [33], this is clearly related to the level of knowledge about the production and characteristics of Iberian meat, which shows that there is a need to increase knowledge in order to give more value to the product and to understand the price. Typically, traditional meat products have a high fat and salt content compared to conventional products [4].

### 3.2. Consumer’s Preferences Concerning the Importance of Certain Attributes When Buying Cured Pork Products from the Black Slavonian Pig

The attributes influencing purchasing decisions when buying cured pork products from the Black Slavonian Pig (production method, producer or brand of cured pork products, quality mark, origin of the product produced in the traditional breeding region of the Black Slavonian Pigs, color, taste, odor of the meat, visible fat content, and percentage of salt) were analyzed on the basis of nine attributes, and consumers’ attitudes were measured on a 5-point importance scale, where the response options ranged from not at all important to extremely important. Based on the respondents’ answers, the basic descriptive statistical indicators (arithmetic mean, median, mode, and standard deviation) were calculated for each attribute, and the results are presented in Table 2. Based on the arithmetic means, it can be concluded that for Croatian consumers, all studied attributes are equally important when purchasing cured pork products. In previous studies conducted with Spanish, French, and British meat, consumers preferred the quality mark, production method, and origin more than other meat attributes [34,35]. According to Saeed et al. [36], taste and other sensory attributes such as aroma and texture remain key indicators of product acceptance and repurchase. This is in line with Salnikova et al. [37], who found in their study that while consumers value origin, traceability, and traditional food, attributes such as manufacturer brand, convenience, and taste are more important. 

Independent *t*-tests and one-way analyses of variance (ANOVA) were used to determine differences between respondents’ individual characteristics. The results of the independent sample *t*-test presented in Table 3 show that there is a significant difference between the variables quality mark (*t* = −2.960, *p* < 0.01), color (*t* = −1.993, *p* < 0.05), odor of meat (*t* = −2.101, *p* < 0.05), and percentage of salt (*t* = −2.040, *p* < 0.05) in relation to the gender of the respondents. Other variables were not found to be statistically significant in relation to the gender of the respondents. The highest mean scores for all attributes were found for female consumers, which means that female consumers pay more attention to the studied attributes when buying cured meat than male consumers. 

The results of an independent *t*-test in Table 4 show that there was a significant difference between the variables origin of cured meat products from Black Slavonian Pig (*t* = 3.430, *p* < 0.01), color (*t* = 2.720, *p* < 0.01), odor (*t* = 2.306, *p* < 0.05), visible fat content (*t* = 2.074, *p* < 0.05), and salt content (*t* = 2.799, *p* < 0.01) with respect to respondents’ knowledge of the breed of pig from which the meat came. Other variables were not found to be statistically significant in terms of knowledge about the breed of pig. The highest mean value of the variables studied was for consumers knowing about the origin of the meat. Fat content is often considered a negative factor, but the presence of fat and intermuscular fat in meat is positively associated with better-tasting, more tender, and juicier meat in Chernukha et al.’s [22] study of Russian consumers. Also, the research by Chernukha et al. [22] concluded that pork from local breeds such as Livny pigs, which differ significantly from the other breeds studied (Duroc, Altai meat breed, and Mangalitsa) in terms of fatty acid composition, can be recommended for functional foods due to their better intramuscular fat content. 

The results presented in Table 5 show significant differences between the socio-demographic characteristics (age of respondents, employment status, number of household members, and number of children under 15 in the household) and the consumption and purchase habits (place of purchase and frequency of consumption of Black Slavonian Pig meat) of the respondents. In the following table, only the statistically significant differences between the variables studied and the selected socio-demographic data and the consumption and purchase habits of the respondents are presented, while the full data can be found in Tables S1–S6 of the Supplementary Material. From the data in Table 5, it can be seen that there are statistically significant differences between the variables percentage salt content in cured meat products from Black Slavonian Pig and age (F = 4.211, *p* < 0.01), employment status of respondents (F = 2.891, *p* < 0.05), number of household members (F = 5.251, *p* < 0.01), and number of children under 15 years in the household (F = 4.958, *p* < 0.01). The percentage of salt in cured meat products from Black Slavonian pig was more important for older (> 55 years) (M = 4.18, SD = 0.756) and retired consumers living alone (M = 4.23, SD = 0.739). Although there were no statistically significant differences with young consumers aged 18–24, the taste of the meat (M = 4.60, SD = 0.531) was most important (Table S1). This is in line with the research of Razmaitė et al. [1], according to which young consumers are less concerned about health and more interested in taste than older consumers. In the study by Török et al. [38], older consumers from Italy rated the production method as important when purchasing cured traditional meat products, while for middle-aged consumers, the visual appearance of the meat was very important. A statistically significant difference was found between the variables producer and brand, quality mark, production method, origin, and visible fat content in cured meat products, as well as the number of children in the household, place of purchase, and frequency of consumption of fresh or cured Black Slavonian Pig meat products. The producer or brand of cured meat products was more important for consumers who have a child under 15 years in the household (M = 3.91, SD = 0.608) and for consumers who buy cured meat products at the butcher’s shop (M = 3.87, SD = 0.808). In contrast, the quality mark was important for consumers with a child (< 15 years) in the household (M = 4.23, SD = 0.685) who buy cured meat products directly from the producer (M = 4.27, SD = 0.868). The production method (M = 4.20, SD = 0.925) and the origin of the meat (M = 4.27, SD = 0.907) were also more important for consumers who buy cured meat products directly from the producer than for consumers who buy cured meat products in a butcher’s shop or supermarket. Consumers who frequently consume fresh or cured meat products place more importance on the variable origin and visible fat content of cured meat products than consumers who never or only occasionally consume Black Slavonian Pig products (Table 5). Studies show that information about the product (origin, production method, color of the meat, fat content) influences consumers’ purchasing decisions and can be used as a differentiation tool in marketing traditional pork products [4,39,40]. Previous studies by Ortega et al. [41] and Balogh et al. [42] show how consumer preferences for pork are influenced by socio-demographic characteristics such as gender, education, and income. Saeed et al. [36] found significant differences between gender and perceived cues for fat, cut, color, and carving, and between respondents’ age and cut, carving, nutritional value, taste, processing method, and purchase motive of processed beef products. 

### 3.3. Segmentation Analysis of Croatian Consumers

Factor analysis was conducted to determine a smaller number of factors representing the interrelationship between nine variables regarding the most important attributes in the purchase of commodities from the Black Slavonian Pig (Table 6). Principal component analysis was applied, considering eigenvalues greater than 1. Varimax rotation with Kaiser normalization was applied to improve the interpretation of factor scores obtained through factor analysis. To confirm that the data were suitable for analysis, the Kaiser–Meyer–Olkin (KMO) measure of sampling adequacy was applied and yielded a value of 0.853, which is very good. Bartlett’s test of sphericity (χ^2^ = 1646.759, *p* < 0.01) also confirmed that the data could be used for factor analysis. Factor analysis revealed two factors (intrinsic and extrinsic quality) that explained 62.6% of the total variance. The calculated Cronbach’s alpha coefficients confirmed the consistency and reliability of the principal component analysis (0.822 to 0.818). According to the data in Table 6, the first factor explained 50.2% of the variance. The variable most strongly associated with the first factor was called intrinsic quality, and this component accounted for the greatest variability of the variance explained. The second factor explained 12.4% of the variance and groups the variables related to the extrinsic quality characteristics of cured meat products. 

Cluster analysis was performed on the basis of the factors resulting from principal component analysis. Through the K-mean cluster analysis, we obtain three clusters. The results of the analysis of variance (ANOVA) show which variables contribute most to the cluster distribution. The F-values show the greatest separation between the clusters. According to the F-values, the variable of extrinsic quality contributed most to the cluster separation (F = 229.774). Table 7 presents the typical characteristics of each cluster. The centers of the final clusters represent the arithmetic means of the individual variables within each final cluster. The first cluster consists of consumers who are more concerned about intrinsic attributes, as they pay relatively high attention to the intrinsic characteristics of cured meat (odor, taste, color, visible fat, and percentage of salt). The second cluster represents consumers who pay less attention to the intrinsic characteristics of cured meat (n = 182). Instead, the extrinsic characteristics of cured meat (production method, origin, producer or brand, and quality mark) are of greatest importance to them, which is why they are called extrinsic consumers. The third and smallest group (n = 105) are rather uninterested or inattentive meat consumers, i.e., they attach below-average importance to the attributes when buying cured meat from Black Slavonian Pig. 

The differences between the clusters were validated by canonical discriminant functions. Discriminant analysis showed that the factors identified had a significant effect (*p* < 0.01) on cluster differentiation. The Wilks’ lambda measures were small (Wilks’ lambda function 1 = 0.232; function 2 = 0.534), and all values associated with the chi-square test (χ^2^) were significant at *p* < 0.01. The results of the discriminant function to classify the importance of certain factors in the purchase of cured pork showed that 99.8% of the initially grouped cases were correctly classified. The market segmentation analysis conducted provides information that can be used in the development of marketing strategies for both producers and retailers. The methodology used in this study is consistent with previous research [42,43,44,45,46,47,48,49,50]. Associations between consumer segments and sociodemographic characteristics of consumers as well as purchasing habits, cured pork consumption habits, and knowledge about the pig breed were determined using the (χ^2^). The results of the chi-square (χ^2^) test are presented in Table 8. They show a lack of significant differences between the three clusters obtained and the socio-demographic characteristics of purchase, consumption habits, and knowledge of the respondents about pig breed. However, the data show that there is a significant dependence between the variables gender (χ^2^ = 9.284, df = 2, *p* < 0.01) and consumers’ knowledge about the pig breed (χ^2^ = 6.066, df = 2, *p* < 0.05) and the obtained clusters.

Of the 123 consumers who formed the intrinsically oriented meat consumers segment, 55.6% were female. This segment is characterized by consumers aged 45–54 and over 55, mainly from central and north-west Croatia. In terms of employment status, employed consumers dominated (64.3%). This segment includes consumers with two to four household members (85.7%), without children under 15 (75.4%), and with an average household income (55.6%). The individuals grouped here turn out to be consumers who purchase cured meat products in the supermarket (50.8%). When they buy cured meat products, they do not know which breed they come from, and they occasionally consume fresh or cured meat products from the Black Slavonian Pig. 

The second segment consists of 182 extrinsically oriented meat consumers, and it was the largest group formed. This segment was dominated by female consumers (55.4%); the most common age range was between 25–34 years (22.6%) and 55 years (29.4%). This segment includes consumers from continental Croatia (central, north-western and eastern Croatia). This segment consists of employed (60.5%) consumers with average household income with two to four household members (79.7%) and four to eight (13.6%) without children under 15. Regarding the characteristics of purchase and consumption shown in Table 8, most consumers in this segment buy cured meat products from a butcher’s shop (51.4%). A total of 77.4% of consumers do not know the breed of the meat they buy, but 22.6% do, and they occasionally consume fresh meat or cured meat products from the Black Slavonian Pig (55.4%).

The smallest third segment (105) of uninterested meat consumers is dominated by men (61.7%), aged 35–44 (24.3%) and 45–54 (23.4%). They are equally represented in all regions, although slightly less in the Northern Adriatic and Lika regions (14.0%) and in the Middle and Southern Adriatic regions (11.2%). This segment consists of employed consumers (61.7%) with an average income (60.7%). The third segment includes consumers with two to four family members in the household (84.1%) without children under 15 (61.7%) and with more than two children under 15 (20.6%) in the household. They buy meat at the supermarket (47.7%) and at the butcher (46.7%). When buying meat, they do not know which breed of pig they are buying meat from (88.8%), and they occasionally consume fresh or cured meat products from the Black Slavonian Pig (56.1%).

The segmentation results show that in the first two segments, which are dominated by female consumers, women pay more attention to intrinsic and extrinsic cues when buying cured meat products than men, who belong to the uninterested meat consumer segment. These findings are supported by previous research results on the importance of intrinsic and extrinsic cues when purchasing meat [51,52,53]. The results also show that consumers lack knowledge about the breed when purchasing cured meat products. It is evident that the consumers who know more about the breed the meat comes from are the consumers of the second segment, who mostly buy cured meat products at the butcher’s shop or from the producers. Consumers who buy meat directly from producers or butchers can obtain more information about the production method, breed, and origin than consumers who buy meat from supermarkets, who lack this information and can only rely on the declaration on the product and the labels, which are usually insufficient. It is important to introduce labels on the Croatian market that provide consumers with transparency and information about the breed, production method, origin, and intrinsic cues. Traditional foods can create strong expectations among consumers about the taste and consumption of the products, and producers can add value to their products through trademarks, Protection of Geographical Indications (PGI), Protected Designations of Origin (PDO), or Guaranteed Traditional Speciality (TSG) [54,55]. Typical Mediterranean-cured meat products are internationally recognized and protected by PDO labels [5]. Traditional products and activities in rural areas have a significant impact on the economic, social, and functional structure of rural regions [6]. In the Republic of Croatia, fresh Black Slavonian Pig meat has recently been protected at the national level with the PDO (Protected Designation of Origin) label (EU level pending) [56]. The research by Jelić Milković et al. [40] on fresh Black Slavonian Pig meat shows that the consumers surveyed derive the greatest utility from the label reared in continental Croatia + PDO and are willing to pay a higher price for fresh Black Slavonian Pig produced outdoors and labeled with the label reared in continental Croatia + PDO. Similar results were found in Italy, where consumers preferred meat with the PGI label [28]. And in the study by Török et al. [55], who conducted a choice experiment with Hungarian consumers to investigate consumer preference for the PGI label compared to leading producer brands, as well as the importance of taste and different price levels. The authors concluded that the PGI label can increase producers’ margins and contribute to local economic development, that the PGI label can add a significant price premium, and that Hungarian consumers in the traditional consumer segment are willing to pay a higher price for a PGI-labelled product, while a minority of consumers in the brand-conscious segment place more value on a private brand. Consumers expect traceability and information to make informed purchasing decisions, and they value PDO and PGI labels, which influence the willingness to pay and substantially add extra value to traditional products [40,55,57].

Before drawing conclusions, we should mention some limitations of the study. Future research should focus on a larger sample of respondents, investigate additional attributes that are relevant to consumers when purchasing cured meat products, and incorporate the psychological characteristics and lifestyle of respondents to create important market segments for the product. Future research should also include a sensory analysis of the cured meat product and a choice experiment to investigate participants’ preferences and assess the economic value or willingness to pay (WTP) for cured meat products from the Black Slavonian Pig.

## 4. Conclusions

The results show that there are significant differences between the nine variables studied and consumers’ socio-demographic characteristics, consumption habits, purchasing behavior, and knowledge about the pig breed. The results showed that gender, knowledge of the pig breed, age, employment status, number of children under 15 years of age, and frequency of consumption of Black Slavonian Pig have a significant influence on consumers’ opinions regarding the variables of production method, quality mark, producer or brand, origin, color, odor, visible fat content, and percentage salt content in cured meta-products. In order to explore consumer preferences in depth in this study, we segmented Croatian consumers according to the most important attributes they consider when purchasing cured meat products from Black Slavonian Pig. The study provides information on the existence of different consumer segments based on attributes that influence consumers’ decisions when purchasing cured meat products in terms of socio-demographic characteristics, general purchasing habits, consumption, and knowledge about the breed. Based on the aforementioned attributes, three consumer segments were identified in this study: intrinsically oriented meat consumers, extrinsically oriented meat consumers, and uninterested meat consumers. The identified segments differ with regard to the socio-demographic variable gender and with regard to the variable knowledge about the pig breed when buying meat. Based on the results, specific marketing strategies can be developed for each consumer segment. It is evident that the Croatian market lacks labels and declarations that provide consumers with more information about traditional cured meat products from the Black Slavonian Pig. Only consumers who know about the breed and frequently consume Black Slavonian Pig meat put more emphasis on specific characteristics of this traditional product, such as origin, visible fat, salt content, and odor, but they also look for quality labels such as labels of Protection of Geographical Indications (PGI), Protected Designations of Origin (PDO), or Guaranteed Traditional Speciality (TSG) that need to be introduced on the Croatian market, as they are perceived by consumers as an important factor. And through which producers and retailers can communicate and share the information consumers are looking for and position themselves in front of different consumer segments, highlighting the unique attributes of traditional products and commanding a premium price.

## Figures and Tables

**Table 1:** Descriptive statistics of the socio-demographic characteristics and consumption habits of the respondents.

		Frequency	Percent (%)
Gender	Male	201	49.0
	Female	209	51.0
Age	18–24	55	13.4
	25–34	84	20.5
	35–44	84	20.5
	45–54	77	18.8
	>55	110	26.8
Region	Central Croatia	102	24.9
	North-western Croatia	101	24.6
	Eastern Croatia	88	21.5
	Northern Adriatic and Lika	65	15.9
	Middle and South Adriatic	54	13.2
Labour situation	Student	45	11.0
	Unemployed	33	8.0
	Employed part-time	17	4.1
	Employed	254	62.0
	Retired	61	14.9
Number of household members	1	26	6.3
	2–4	339	82.7
	5–8	45	11.0
Number of children below 15 years in the household	0	282	68.8
	1	70	17.1
	≥2	58	14.1
Household income group	Significantly below average	12	2.9
	Below average	70	17.1
	Average	241	58.8
	Above average	87	21.2
Usual place of purchase	Direct from producer	30	7.3
	Butcher shop	197	48.0
	Hyper/supermarket	183	44.6
Knowledge about the breed of pig from which the meat originates	Yes	73	17.8
	No	337	82.2
Consumption frequency of fresh meat or cured products from Black Slavonian Pig	Never	166	40.5
	Yes, occasionally	226	55.1
	Yes, frequently	18	4.4

**Table 2:** Consumer opinions on the importance of certain attributes When buying cured pork products from the Black Slavonian Pig.

	M	M_e_	M_o_	SD
Taste of the meat	4.48	5	5	0.614
Odor of the meat	4.44	5	5	0.673
Color of the meat	4.16	4	4	0.690
Quality mark	4.12	4	4	0.742
Origin (regions of the Republic of Croatia, autochthonous breed)	4.07	4	4	0.788
Production method (domestic or industrial production)	4.06	4	4	0.714
Visible fat content	4.00	4	4	0.750
Percentage of salt	3.93	4	4	0.836
Producer or brand	3.75	4	4	0.828

Note: M—arithmetic mean; M_e_—median; M_o_—mode; SD—standard deviation.

**Table 3:** Testing the differences in the mean values of the variables in relation to the gender of the respondents.

Variables	Female		Male		*t*-Test	*p*
	M	SD	M	SD		
Production method (domestic or industrial production)	4.12	0.714	4.00	0.711	−1.771	0.077
Producer or brand	3.80	0.819	3.70	0.837	−1.193	0.234
Quality mark	4.22	0.659	4.01	0.806	−2.960	0.003 **
Origin (regions of the Republic of Croatia, autochthonous breed)	4.11	0.767	4.03	0.809	−0.966	0.334
Color of the meat	4.22	0.630	4.09	0.743	−1.993	0.047 *
Taste of the meat	4.53	0.580	4.42	0.644	−1.788	0.074
Odor of the meat	4.51	0.644	4.37	0.696	−2.101	0.036 *
Visible fat content	4.04	0.723	3.96	0.777	−1.186	0.236
Percentage of salt	4.01	0.743	3.85	0.917	−2.040	0.042 *

Note: M—arithmetic mean; SD—standard deviation; ** *p* < 0.01; * *p* < 0.05.

**Table 4:** Testing the differences in the mean values of the variables related to the respondents’ knowledge of the breed of pig from which the meat originates.

Variables	Yes		No		*t*-Test	*p*
	M	SD	M	SD		
Production method (domestic or industrial production)	4.21	0.763	4.03	0.700	1.946	0.052
Producer or brand	3.92	0.909	3.72	0.807	1.901	0.058
Quality mark	4.26	0.708	4.09	0.747	1.793	0.074
Origin (regions of the Republic of Croatia)	4.36	0.653	4.01	0.802	3.430	0.001 **
Color of the meat	4.36	0.632	4.12	0.695	2.720	0.007 **
Taste of the meat	4.58	0.551	4.46	0.626	1.495	0.136
Odor of the meat	4.60	0.571	4.40	0.688	2.306	0.022 *
Visible fat content	4.16	0.764	3.96	0.743	2.074	0.039 *
Percentage of salt	4.18	0.788	3.88	0.838	2.799	0.005 **

Note: M—arithmetic mean; SD—standard deviation; ** *p* < 0.01; * *p* < 0.05.

**Table 5:** Testing the differences in the mean values of the variables in relation to socio-demographic and consumption habits.

	**18–24**		**25–34**		**35–44**		**45–54**		**>55**		***p***
	M	SD	M	SD	M	SD	M	SD	M	SD	
Percentage of salt	3.67	0.862	3.85	0.703	3.89	0.905	3.90	0.912	4.18	0.756	0.002 **
	**Student**		**Unemployed**		**Employed part-time**		**Employed**		**Retired**		***p***
	M	SD	M	SD	M	SD	M	SD	M	SD	
Percentage of salt	3.71	0.869	3.91	0.947	3.82	0.728	3.91	0.831	4.23	0.739	0.022 *
					**1**		**2–4**		**5–8**		***p***
					M	SD	M	SD	M	SD	
Percentage of salt					4.42	0.578	3.91	0.849	3.80	0.786	0.006 **
					**0**		**1**		**≥2**		***p***
					M	SD	M	SD	M	SD	
Producer or brand					3.76	0.814	3.91	0.608	3.53	1.063	0.035 *
Quality mark					4.14	0.700	4.23	0.685	3.90	0.949	0.031 *
Percentage of salt					4.02	0.798	3.71	0.783	3.78	1.009	0.007 **
					**Direct from producer**		**Butcher shop**		**Hyper/supermarket**		***p***
					M	SD	M	SD	M	SD	
Production method					4.20	0.925	4.13	0.706	3.96	0.674	0.042 *
Producer or brand					3.77	0.858	3.87	0.808	3.62	0.829	0.011 *
Quality mark					4.27	0.868	4.23	0.680	3.98	0.763	0.002 **
Origin					4.27	0.907	4.19	0.758	3.91	0.772	0.001 **
					**Never**		**Yes, occasionally**		**Yes, frequently**		***p***
					M	SD	M	SD	M	SD	
Origin					3.96	0.781	4.13	0.792	4.44	0.616	0.013 *
Visible fat content					3.92	0.742	4.02	0.751	4.50	0.618	0.006 **

Note: M—arithmetic mean; SD—standard deviation; ** *p* < 0.01; * *p* < 0.05; Other variables did not prove to be statistically significant with chosen sociodemographic and consumption and purchasing habits, but results are presented in the Supplementary Materials in Tables S1–S6.

**Table 6:** Consumer attitudes towards the importance of certain attributes when buying cured products from the Black Slavonian Pig.

	F1	F2
Odor of the meat	**0.857**	0.143
Taste of the meat	**0.836**	0.153
Color of the meat	**0.662**	0.371
Visible fat content	**0.652**	0.339
Percentage of salt	**0.546**	0.380
Producer or brand	0.077	**0.806**
Quality mark	0.330	**0.747**
Origin (regions of the Republic of Croatia)	0.316	**0.733**
Production method (domestic or industrial production)	0.325	**0.731**
*% Variance Explained*	50.270	12.351
*Eigenvalues*	4.524	1.112
*Cronbach’s Alpha*	0.822	0.818

KMO = 0.853, Bartlett’s χ^2^ = 1646.759, *p* = 0.000. Extraction Method: Principal Component Analysis. Rotation Method: Varimax with Kaiser Normalization. Note: intrinsic quality (F1); extrinsic quality (F2).

**Table 7:** Final cluster profiles regarding attributes when buying cured meat from the Black Slavonian Pig.

	Custer 1 (n = 123) Intrinsically Oriented Meat Consumers	Cluster 2 (n = 182) Extrinsically Oriented Meat Consumers	Cluster 3 (n = 105) Uninterested Meat Consumers
Intrinsic quality	0.91445	−0.05907	−0.96883
Extrinsic quality	−0.66815	0.81817	−0.63547

**Table 8:** Consumer segments according to their socio-demographic characteristics: purchasing, consumption habits, and knowledge of respondents (χ^2^-test).

		Cluster (Consumers %)				
		C1	C2	C3	χ^2^	*p*
Gender	Male	44.4	44.6	61.7	9.284	0.010 **
	Female	55.6	55.4	38.3		
Age	18–24	11.1	14.7	14.0	8.961	0.346
	25–34	19.0	22.6	18.7		
	35–44	19.0	19.2	24.3		
	45–54	21.4	14.1	23.4		
	>55	29.4	29.4	19.6		
Region	Central Croatia	30.2	21.5	24.3	15.271	0.054
	North-western Croatia	31.0	20.3	24.3		
	Eastern Croatia	14.3	23.7	26.2		
	Northern Adriatic and Lika	15.9	16.9	14.0		
	Middle and South Adriatic	8.7	17.5	11.2		
Labour status	Student	7.9	13.0	11.2		
	Unemployed	11.1	4.5	10.3		
	Employed part-time	4.0	3.4	5.6	10.628	0.224
	Employed	64.3	60.5	61.7		
	Retired	12.7	18.6	11.2		
Number of household members	1	7.1	6.8	4.7	3.781	0.436
	2–4	85.7	79.7	84.1		
	5–8	7.1	13.6	11.2		
Number of children in household (<15)	0	75.4	68.4	61.7	7.999	0.092
	1	12.7	19.8	17.8		
	≥2	11.9	11.9	20.6		
Household income group	Significantly below average	5.6	2.3	0.9	8.533	0.202
	Below average	14.3	16.4	21.5		
	Average	55.6	59.9	60.7		
	Above average	24.6	21.5	16.8		
Usual place of purchase	Direct from producer	4.8	10.2	5.6	7.139	0.129
	Butcher shop	44.4	51.4	46.7		
	Hyper/supermarket	50.8	38.4	47.7		
Knowledge about the breed of pig	Yes	16.7	22.6	11.2	6.066	0.048 *
	No	83.3	77.4	88.8		
Consumption frequency of fresh meat or cured products from Black Slavonian Pig	Never	41.3	38.4	43.0	4.714	0.318
	Yes, occasionally	54.0	55.4	56.1		
	Yes, frequently	4.8	6.2	0.9		

Note: ** *p* < 0.01, * *p* < 0.05; intrinsic cue concerned meat consumers (C1); extrinsic cue concerned meat consumers (C2); uninterested meat consumers (C3).

## Data Availability

The data obtained in the experiment can be retrieved from the corresponding authors upon reasonable request.

## Supplementary Materials

The following supporting information can be downloaded at: https://www.mdpi.com/article/10.3390/foods12193666/s1. Table S1: Testing the differences in the mean values of the variables in relation to the respondent’s age; Table S2: Testing the differences in the mean values of the variables in relation to the respondent’s labor situation; Table S3: Testing the differences in the mean values of the variables in relation to the number of household members; Table S4: Testing the differences in the mean values of the variables in relation to the number of children under 15 years in the household; Table S5: Testing the differences in the mean values of the variables related to the place of purchase of cured pork products from the Black Slavonian Pig; Table S6: Testing the differences in the mean values of the variables related to the consumption frequency of fresh meat or cured products from the Black Slavonian Pig.
