# Supplementary material for: Consumer Preferences for Cured Meat Products from the Autochthonous Black Slavonian Pig
Source: Foods. 2023 Oct 5;12(19):3666. doi: 10.3390/foods12193666 (PMC10572601; doi:10.3390/foods12193666)
Supplement: Supplementary file 1 [file foods-12-03666-s001.zip › foods-2640526-supplementary.pdf]

Table S1. Testing the differences in the mean values of the variables in relation to the respondent's age

Variables	18 – 24		25 – 34		35 – 44		45 – 54		> 55		*F*	*p*
	M	SD	M	SD	M	SD	M	SD	M	SD		
Production method	4.15	0.705	4.13	0.673	3.99	0.685	3.99	0.734	4.06	0.758	0.817	0.515
Producer or brand	3.85	0.756	3.75	0.758	3.64	0.914	3.75	0.797	3.78	0.871	0.609	0.657
Quality mark	4.16	0.788	4.10	0.738	4.02	0.776	4.12	0.725	4.19	0.71	0.673	0.611
Origin	4.18	0.748	4.12	0.735	4.05	0.775	4.00	0.858	4.05	0.811	0.534	0.711
Color of the meat	4.20	0.730	4.05	0.71	4.15	0.668	4.09	0.692	4.27	0.662	1.539	0.190
Taste of the meat	4.60	0.531	4.40	0.583	4.42	0.585	4.53	0.661	4.48	0.66	1.205	0.308
Odor of the meat	4.35	0.865	4.37	0.655	4.37	0.617	4.51	0.641	4.55	0.63	1.613	0.170
Visible fat content	3.91	0.727	3.87	0.690	4.06	0.75	3.94	0.817	4.15	0.74	2.179	0.071
Percentage of salt	3.67	0.862	3.85	0.703	3.89	0.905	3.90	0.912	4.18	0.756	4.211	0.002**

Note: M—arithmetic mean; SD—standard deviation; ** $p < 0.01$; * $p < 0.05$.

Table S2. Testing the differences in the mean values of the variables in relation to the respondent's labor situation

Variables	Student		Unemployed		Employed part-time		Employed		Retired		*F*	*p*
	M	SD	M	SD	M	SD	M	SD	M	SD		
Production method	4.11	0.832	3.94	0.556	3.94	0.748	4.07	0.722	4.08	0.666	0.428	0.788
Producer or brand	3.84	0.928	3.52	0.906	3.59	0.712	3.76	0.829	3.80	0.726	1.052	0.380
Quality mark	4.09	0.925	3.88	0.820	4.12	0.697	4.14	0.734	4.20	0.572	1.092	0.360
Origin	4.16	0.824	3.82	0.846	3.76	0.752	4.08	0.793	4.20	0.679	2.044	0.087
Color of the meat	4.22	0.850	4.15	0.712	3.94	0.556	4.14	0.683	4.26	0.603	0.920	0.452
Taste of the meat	4.47	0.726	4.61	0.496	4.35	0.702	4.48	0.614	4.44	0.563	0.588	0.672
Odor of the meat	4.24	1.004	4.55	0.564	4.47	0.717	4.44	0.631	4.49	0.566	1.260	0.285
Visible fat content	3.87	0.869	4.03	0.770	3.71	1.047	4.01	0.708	4.11	0.709	1.401	0.233
Percentage of salt	3.71	0.869	3.91	0.947	3.82	0.728	3.91	0.831	4.23	0.739	2.891	0.022*

Note: M—arithmetic mean; SD—standard deviation; ** $p < 0.01$; * $p < 0.05$.

Table S3. Testing the differences in the mean values of the variables in relation to the number of household members

Variables	1		2 - 4		5 - 8		*F*	*p*
	M	SD	M	SD	M	SD		
Production method	4.15	0.881	4.05	0.699	4.09	0.733	0.314	0.731
Producer or brand	3.96	0.871	3.75	0.809	3.62	0.936	1.387	0.251
Quality mark	4.23	0.652	4.11	0.713	4.11	0.982	0.311	0.733
Origin	4.23	0.652	4.04	0.793	4.24	0.802	1.924	0.147
Color of the meat	4.15	0.675	4.17	0.690	4.11	0.714	0.122	0.885
Taste of the meat	4.54	0.508	4.49	0.603	4.33	0.739	1.474	0.230
Odor of the meat	4.50	0.583	4.45	0.662	4.29	0.787	1.318	0.269
Visible fat content	4.27	0.533	4.00	0.750	3.84	0.824	2.665	0.071
Percentage of salt	4.42	0.578	3.91	0.849	3.80	0.786	5.251	0.006**

Note: M—arithmetic mean; SD—standard deviation; ** $p < 0.01$; * $p < 0.05$.

Table S4. Testing the differences in the mean values of the variables in relation to the number of children under 15 years in the household

Variables	0		1		≥ 2		*F*	*p*
	M	SD	M	SD	M	SD		
Production method	4.08	0.709	4.04	0.6	3.97	0.858	0.654	0.520
Producer or brand	3.76	0.814	3.91	0.608	3.53	1.063	3.384	0.035*
Quality mark	4.14	0.700	4.23	0.685	3.9	0.949	3.508	0.031*
Origin	4.09	0.766	4.09	0.756	3.98	0.927	0.444	0.642
Color of the meat	4.18	0.688	4.19	0.644	4.03	0.748	1.097	0.335
Taste of the meat	4.51	0.586	4.43	0.604	4.4	0.748	1.053	0.350
Odor of the meat	4.47	0.643	4.37	0.705	4.36	0.765	1.065	0.346
Visible fat content	4.03	0.742	4.01	0.648	3.83	0.881	1.808	0.165
Percentage of salt	4.02	0.798	3.71	0.783	3.78	1.009	4.958	0.007**

Note: M—arithmetic mean; SD—standard deviation; ** $p < 0.01$; * $p < 0.05$.

Table S5. Testing the differences in the mean values of the variables related to the place of purchase of cured pork products from the Black Slavonian Pig

Variables	Direct from producer		Butcher shop		Hyper/supermarket		*F*	*p*
	M	SD	M	SD	M	SD		
Production method	4.20	0.925	4.13	0.706	3.96	0.674	3.206	0.042*
Producer or brand	3.77	0.858	3.87	0.808	3.62	0.829	4.602	0.011*
Quality mark	4.27	0.868	4.23	0.680	3.98	0.763	6.188	0.002**
Origin	4.27	0.907	4.19	0.758	3.91	0.772	7.195	0.001**
Color of the meat	4.13	0.860	4.19	0.688	4.13	0.663	0.471	0.625
Taste of the meat	4.37	0.850	4.53	0.549	4.44	0.634	1.450	0.236
Odor of the meat	4.47	0.860	4.47	0.674	4.4	0.638	0.438	0.646
Visible fat content	3.97	0.890	4.04	0.762	3.96	0.714	0.555	0.574
Percentage of salt	4.10	0.923	4.00	0.776	3.83	0.876	2.622	0.074

Note: M—arithmetic mean; SD—standard deviation; ** $p < 0.01$; * $p < 0.05$.

Table S6. Testing the differences in the mean values of the variables related to the consumption frequency of fresh meat or cured products from the Black Slavonian Pig

Variables	Never		Yes, occasionally		Yes, frequently		*F*	*p*
	M	SD	M	SD	M	SD		
Production method	4.03	0.674	4.07	0.718	4.22	1.003	0.617	0.540
Producer or brand	3.71	0.802	3.76	0.837	4.06	0.938	1.420	0.243
Quality mark	4.08	0.713	4.14	0.768	4.28	0.669	0.728	0.483
Origin	3.96	0.781	4.13	0.792	4.44	0.616	4.404	0.013*
Color of the meat	4.16	0.669	4.14	0.714	4.44	0.511	1.660	0.191
Taste of the meat	4.47	0.558	4.46	0.66	4.78	0.428	2.267	0.105
Odor of the meat	4.43	0.596	4.42	0.734	4.72	0.461	1.676	0.188
Visible fat content	3.92	0.742	4.02	0.751	4.50	0.618	5.256	0.006**
Percentage of salt	3.90	0.833	3.92	0.821	4.28	1.018	1.648	0.194

Note: M—arithmetic mean; SD—standard deviation; ** $p < 0.01$; * $p < 0.05$.
